# Achalasia and Cricopharyngeal Sphincter Dysfunction in a Patient With Myasthenia Gravis: A Case Report

**DOI:** 10.7759/cureus.42575

**Published:** 2023-07-27

**Authors:** Sudeep Acharya, Shamsuddin Anwar, Kumar Thapa, Rabih Maroun, Thomas M Kilkenny

**Affiliations:** 1 Internal Medicine/Pulmonary and Critical Care Medicine, Donald and Barbara Zucker School of Medicine at Hofstra/Northwell, Staten Island, USA; 2 Infectious Diseases, Tufts Medical Center, Boston, USA; 3 Internal Medicine, Staten Island University Hospital, Staten Island, USA; 4 Pulmonary and Critical Care Medicine, Staten Island University Hospital, Staten Island, USA; 5 Sleep Medicine, Staten Island University Hospital, Staten Island, USA

**Keywords:** cricopharyngeal dysfunction, pulmonary critical care, achalasia cardia, oropharyngeal dysphagia, myasthenia gravis (mg)

## Abstract

This case report describes an 82-year-old female patient with myasthenia gravis (MG) who presented with worsening dysphagia. The patient was found to have cricopharyngeal sphincter and esophageal achalasia, and a percutaneous endoscopic gastrostomy (PEG) tube was placed due to severe pharyngeal dysphagia and cricopharyngeal dysfunction. The patient had class IVb myasthenia gravis and was treated with intravenous immunoglobulin (IVIG), prednisone, and pyridostigmine. The report discusses the link between myasthenia gravis and dysphagia, which is seen in 20% of patients. The report also explores the relationship between myasthenia gravis and achalasia, which is a rare disorder characterized by the failure of relaxation of the sphincter muscles. While myasthenia gravis leads to muscle weakness and should not cause achalasia, there have been a few case reports describing a link between the two disorders. Cricopharyngeal dysfunction, which is a common disorder causing dysphagia in the elderly, was also noted in the patient. The report highlights that cricopharyngeal dysfunction may be primary or secondary, with the latter often being associated with inflammatory myopathies such as polymyositis or mixed connective tissue disorders. The patient did not have a workup for other autoimmune diseases that could have been the cause of achalasia.

## Introduction

Myasthenia gravis (MG) is an autoimmune disorder of neuromuscular junction that often presents with proximal muscle weakness and ocular muscle weakness. Isolated dysphagia is a less common initial presentation and is usually related to bulbar musculature. Achalasia as the cause of dysphagia has been rarely described in patients with myasthenia gravis, and the exact mechanism is unknown [1,2]. Here, we present a case of cricopharyngeal sphincter dysfunction and esophageal achalasia requiring percutaneous endoscopic gastrostomy (PEG) tube placement in a patient diagnosed with myasthenia gravis.

## Case presentation

**Figure 1:**
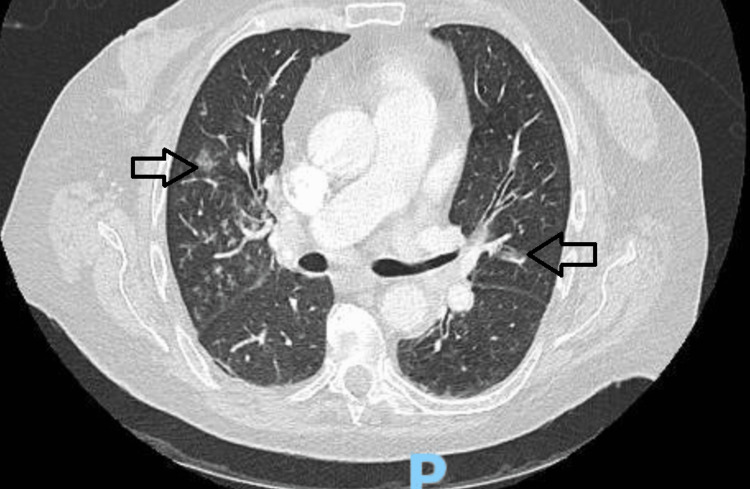
Computed tomography of the chest Bilateral patchy nodular areas of ground-glass opacifications (arrowheads) in the lung parenchyma.

An 82-year-old female with a past medical history of diabetes mellitus (well-controlled without a history of neuropathy), hypertension, and dyslipidemia presented to our facility for evaluation for dysphagia worsening over one week. The dysphagia was initially with solid foods but progressed rapidly for liquids by the time of presentation. A chest computed tomography (CT) was performed, which revealed mediastinal lymphadenopathy, numerous patchy ground-glass opacities, and a multinodular thyroid gland with no evidence of airway obstruction (Figure 1). CT of the head was negative for any intracranial pathology.

The patient underwent upper endoscopy, which showed an esophageal diverticulum in the distal esophagus about 5 centimeters proximal to the gastroesophageal junction and mild stenosis of the lower esophageal sphincter causing mild resistance to the passage of the scope. Additional findings of spastic contractility and a proximal dilation in the esophagus along with a watermelon mucosal appearance were noted. These findings were suggestive of a dysmotility disorder with a possible etiology of achalasia. Biopsies were obtained to rule out eosinophilic esophagitis.

The patient was also found to have severe pharyngeal dysphagia given signs of overt aspiration at bedside upon evaluation by speech and swallow. On neurological evaluation, there was a significant reduction in palatal elevation and hypernasal voice, worse in the upright position, which was suggestive of the involvement of the vagus nerve. Given bulbar weakness, serum acetylcholine receptor antibodies were sent to test for myasthenia gravis.

The patient’s hospital course was complicated by severe aspiration pneumonia requiring a mechanical ventilator for four days. She had persistent severe dysphagia post-extubation. Meanwhile, a repeat speech and swallow evaluation with videofluoroscopy re-demonstrated severe obstruction of flow through the pharyngoesophageal segment and severe cricopharyngeal dysfunction.

Due to the severity of the symptoms, the patient underwent percutaneous endoscopic gastrostomy (PEG) tube placement. Finally, the acetylcholine receptor antibodies came back positive, confirming the diagnosis of myasthenia gravis. The patient was initiated on treatment with immunoglobulin, prednisone, and pyridostigmine. Significant improvement in dysphagia was noted after the treatment.

## Discussion

Myasthenia gravis (MG) is an uncommon autoimmune disorder of neuromuscular junction mediated by antibodies against acetylcholine receptors. It can be characterized by fluctuating weakness involving ocular, bulbar, limb, or respiratory muscles and varying patterns. According to an estimate, MG affects 3.2 per 100,000 patients in the United States with variable (15%-40%) patients presenting with dysphagia [1,2]. These symptoms were grossly absent except for dysphagia in our case, which was interesting to note.

Bulbar weakness causing dysphagia and dysarthria can be a presentation for myasthenia gravis, but achalasia is an uncommon and rare finding [3-5]. There are only a few case reports noted in the medical literature describing the association of myasthenia gravis with achalasia, often described as a paraneoplastic phenomenon with concomitant diagnosis of thymoma (unlike our case where no evidence of thymoma was noted) [6,7]. The symptoms include failure of relaxation or spasm of the cricopharyngeal muscles as well as improper relaxation of sphincter muscles of the gastroesophageal junction resulting in achalasia. Our patient’s chest imaging did not reveal any evidence of thymoma. Given the rarity of the condition and association, the exact diagnosis was delayed in our case. The main therapeutic modalities for myasthenia gravis include steroids and other immunosuppressants, along with immunoglobulin and cholinergic agents [8]. With appropriate treatment, the symptoms of achalasia are expected to improve as noted in our case. In the presence of a thymoma, surgical resection of the thyroid resection is often indicated [9].

## Conclusions

Myasthenia gravis usually presents with weakness of ocular and proximal muscles. Dysphagia is a less common presentation. There is limited medical literature to describe myasthenia gravis linked with achalasia, and the underlying mechanism is unknown. Patients’ dysphagia and achalasia while being investigated for autoimmune disorders should also be evaluated for myasthenia gravis in differential diagnosis. Treatment should be directed toward the underlying disease process.
